# Impact of COVID-19 on the Pediatric Primary Care Model in Catalonia: Analysis of Changes in the Number and Type of Face-to-Face and Remote Visits

**DOI:** 10.2196/49943

**Published:** 2024-03-15

**Authors:** Marta Castillo-Rodenas, José Ángel Vicente Gómez, Aïna Fuster-Casanovas, Queralt Miró Catalina, Josep Vidal-Alaball, Francesc López Seguí

**Affiliations:** 1 Centre d'Atenció Primària Cardona, Gerència d'Atenció Primària i a la Comunitat Catalunya Central, Institut Català de la Salut, Cardona, Spain; 2 Centre de Recerca en Economia de la Salut, Universitat Pompeu Fabra, Barcelona, Spain; 3 Unitat de Suport a la Recerca de la Catalunya Central, Fundació Institut Universitari per a la Recerca a l'Atenció Primària de Salut Jordi Gol i Gurina, Sant Fruitós de Bages, Spain; 4 Health Promotion in Rural Areas Research Group, Gerència d'Atenció Primària i a la Comunitat Catalunya Central, Institut Català de la Salut, Sant Fruitós de Bages, Spain; 5 Faculty of Medicine, University of Vic - Central University of Catalonia (UVic-UCC), Vic, Spain; 6 Chair in ICT and Health, Centre for Health and Social Care Research (CESS), University of Vic - Central University of Catalonia (UVic-UCC), Vic, Spain

**Keywords:** COVID-19, remote consultation, primary health care, digital health, pediatric, face-to-face, telemedicine

## Abstract

**Background:**

The outbreak of COVID-19 has turned the care model of health systems around the world upside down. The health care crisis has led to opportunities for digital health to deliver quality care, and the system has been redirected toward telemedicine. In Catalonia, Spain, as of March 2020, the pattern of visits in primary care pediatric consultations changed, such that face-to-face visits decreased in favor of non–face-to-face visits.

**Objective:**

This study aimed to analyze variations in the types of pediatric visits in primary care centers in Catalonia before and after the onset of COVID-19.

**Methods:**

This was a descriptive observational study based on administrative data. The number and type of visits to primary care pediatric services in Catalonia between January 2019 and December 2022 were studied.

**Results:**

A drop of more than 80% in face-to-face visits and an increase of up to 15 times in remote visits were observed as of March 2020 compared to the previous year. Subsequently, the face-to-face attendance rate began to recover, although it did not reach the same rate as before COVID-19. Non–face-to-face visits were maintained, representing more than 20% of the total after more than 2 years of the pandemic.

**Conclusions:**

COVID-19 has been the trigger for a transition in the types of visits to primary care pediatric services. The COVID-19 pandemic was a clear catalyst for the integration of telemedicine in Catalan pediatric health care. In this context, although face-to-face consultations have recovered in absolute numbers, after the pandemic period, the weight of telemedicine has increased.

## Introduction

The COVID-19 pandemic has turned the care model of health systems around the world upside down, causing a significant and often unexpected transformation or disruption in the way health care services are delivered and organized worldwide [1]. In Catalonia, Spain, the health care crisis has led to opportunities for digital health to deliver quality care, and the system has been redirected toward telemedicine [2,3].

Both primary care (PC) centers and hospitals in Catalonia and in many other countries had to adapt to the new situation, where in the face of uncertainty, people (including children) were recommended to not go to health centers in person unless strictly necessary [4,5]. Therefore, as of March 2020, the pattern of visits changed in PC pediatric consultations [6,7]. As with all other services, face-to-face consultations decreased considerably while non–face-to-face consultations increased significantly worldwide [8,9]. In Spain, the reduction in visits during the first months of the declaration of the state of alarm mainly affected child health program check-ups and acute demand due to infectious diseases [10]. In general terms, the acute infections that decreased the most once the strictest confinement started were respiratory and digestive infections [11]. At the same time, telephone consultations multiplied considerably. Later, in June 2020, despite the expected difficulties due to the fear of users and health care workers and the barrier of accessibility to PC, face-to-face attendance was not as low, as health care was managed more appropriately [12].

A multicenter study coordinated by the Spanish Association of Primary Care Paediatrics in June 2020 explains how PC pediatric services were organized during the first wave of COVID-19 and indicates that, in general, pediatricians followed the center’s own contingency plan and that total consultations reduced by approximately 40% to 50% compared to the same period of the previous year [13]. There was a very significant increase in the rate of teleconsultations, such that more than 90% of pediatricians made teleconsultations as opposed to 38% who did so before the pandemic. In contrast, a decrease in on-site visits was noted, and these were mostly (88% of the cases) visits for the child health program.

In Catalonia, an autonomous community of the Spanish state with full competence in health matters, public health care coverage is guaranteed to all citizens through almost 400 PC centers. In pediatrics, in addition to visits for acute and chronic diseases and follow-up, the child health program called *Infància amb Salut* [14] is applied. It is a protocol developed by the Public Health Agency of Catalonia and includes actions that are carried out individually by the PC pediatric team for preventive visits and check-ups, scheduled between 0 and 14 years of age. It includes screenings, vaccines, and health education. It is implemented throughout the public health network of Catalonia. In this way, access to a high-quality child health service is guaranteed universally, that is, for all children and adolescents residing in Catalonia regardless of their socioeconomic situation or place of residence. These visits are called child health check-ups and have specific characteristics, which are different from the visits carried out for acute or chronic diseases or follow-ups.

A previous analysis conducted in Catalonia suggests that non–face-to-face visits (telephone and teleconsultation) increased threefold during the pandemic, while face-to-face visits fell by almost half. Despite this, there are no specific data on the evolution of the pattern of PC pediatric visits throughout the COVID-19 pandemic [15]. In this context, the objective of this study is to describe the quantitative evolution of different types of visits, both remote or electronic and on-site, from 2019 to 2022 in PC pediatric services in Catalonia.

## Methods

### Study Type

This was a retrospective, descriptive, and observational study in the Autonomous Community of Catalonia, Spain.

### Population, Place, and Study Period

The study population comprised a sample of all the children who resided in Catalonia and accessed services between January 1, 2019, and December 31, 2022. This extended study period allowed for the collection of comprehensive data on the demand of child health care services over a period of 4 years.

Data were collected from the Information System of Information Technologies of the Primary Care Services of Catalonia database belonging to the Catalan Health Institute. Data regarding the number and type of visits between January 2020 and December 2022 were analyzed and compared to the corresponding period in the previous year (2019; ie, before the pandemic).

### Variables

Three variables were considered: date of visit, number of visits in a given day, and type of visit; the latter is a categorical variable with 7 types of visits that are established in pediatric PC in Catalonia (9C, 9R, 9D, 9T, 9Ev, 9E, and 9Ec), which were reworked into 3 groups: face-to-face (9C, 9R, and 9D), synchronous non–face-to-face (9T and 9Ev), and asynchronous non–face-to-face (9E and 9Ec) visits. Table 1 summarizes the particularities of each visit type in relation to face-to-face attendance, duration, and synchronicity.

**Table 1.** Characteristics of the types of visits to pediatric primary care in Catalonia.

Designation	Types of visits	Face-to-face attendance	Average duration (min)	Synchronicity
9C	Spontaneous medical appointments	Yes	12	Yes
9R	Child health program check-up	Yes	20	Yes
9D	Home visit	Yes	30	Yes
9T	Telephone consultation	No	6	Yes
9Ev	Video call consultation	No	12	Yes
9Ec	Consultation through the eConsultation digital platform	No	6	No
9E	Non–face-to-face consultation (report, prescription, etc)	No	6	No

There are 3 types of face-to-face visits: short visits dedicated practically to acute demand (9C), child health program check-ups (9R), and home visits (9D). Synchronous non–face-to-face visits include telephone consultations (9T) and video call consultations (9Ev). Asynchronous non–face-to-face consultations comprise eConsultations (9Ec) through La Meva Salut, a personal digital health space, and non–face-to-face consultations (9E), which include consultations where the health care professional does not have direct contact with the user—often because it has been managed through administrative staff, involving procedures such as medication plan preparation, report writing, test evaluation, data management, interconsultation between professionals, and coordination with other services.

### Statistical Analysis

To describe variables, we expressed continuous variables as means and SDs, and we summarized categorical variables as percentages. As for the *t* tests, a Welch unequal variances (2-tailed) *t* test was carried out to compare the mean of total pediatric visits made each month for 2019, 2020, 2021, and 2022, to check if there had been a recovery of total visits. Then, the same Welch test was performed for each group (face-to-face, remote synchronous, and remote asynchronous). Bonferroni correction has been applied to all *P* values. All statistical analyses were conducted using R software (version 4.1.0; R Foundation for Statistical Computing), and the significance level was set at 5%. To smooth the plots, a 14-day rolling average was performed.

### Ethical Considerations

No ethical approval was required, as analyses were conducted only on aggregated data.

## Results

### General Evolution of Visits to PC Pediatric Services

**Figure 1.**
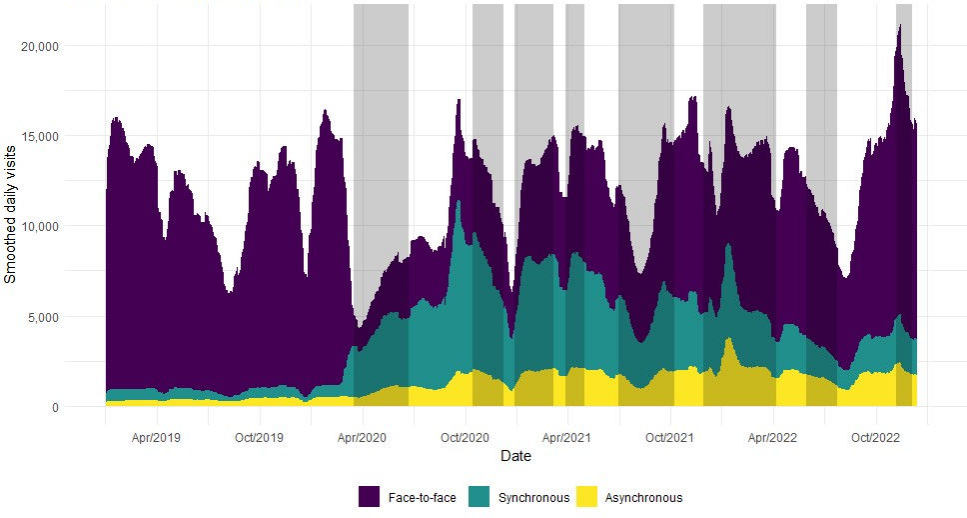
Average number of daily visits by type (face-to-face, synchronous non–face-to-face, and asynchronous non–face-to-face) to pediatric primary care in Catalonia between January 2019 and December 2022. The vertical stripes indicate the 8 waves of COVID-19 in Catalonia.

Figure 1 [16,17] shows the evolution of the average number of daily visits made during the study period, between January 2019 and December 2022, to PC pediatric services in Catalonia, grouped according to the type of visit. The vertical stripes indicate the 8 waves of COVID-19 that can be defined in Catalonia with data from the Statistical Institute of Catalonia and the Information System for the Surveillance of Infections in Catalonia (*Sistema d’Informació per a la Vigilància d’Infeccions a Catalunya*; SIVIC) [16,17]. It is evident how during 2019, the volume of activity in PC pediatric services was predominantly through face-to-face visits and that this pattern changed completely after COVID-19.

**Figure 2.**
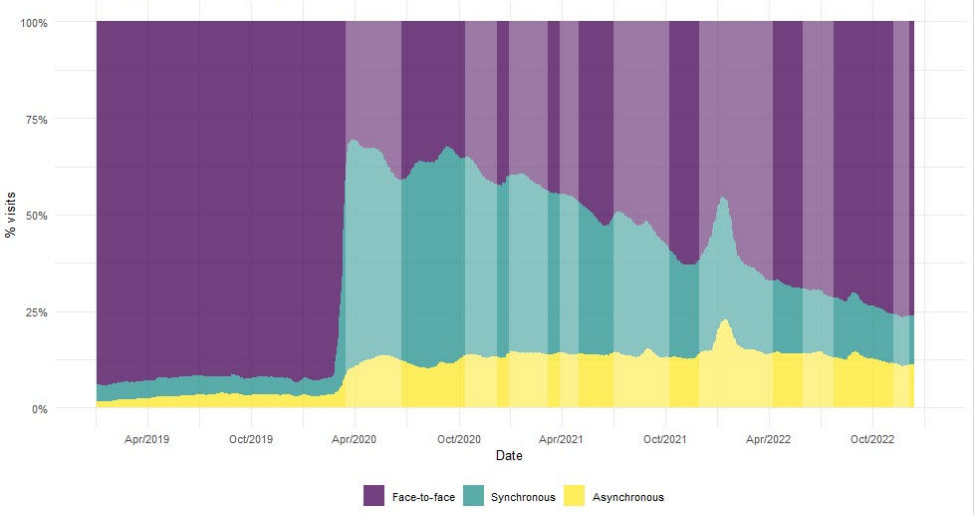
Distribution of daily visits by type (face-to-face, synchronous non–face-to-face, and asynchronous non–face-to-face) to pediatric primary care in Catalonia between January 2019 and December 2022. The vertical stripes indicate the 8 waves of COVID-19 in Catalonia.

Figure 2 [16,17] shows the distribution of the 3 types of visits to PC pediatric services in Catalonia over the last 3 years. The drop in the relative weight of face-to-face visits with the outbreak of the COVID-19 pandemic is noteworthy. Later, they slowly picked up again but did not reach the 2019 proportion at the end of the study. Figure 2 shows that face-to-face visits had recovered in absolute numbers but not in proportion because telemedicine consultation had increased its weight. In parallel, synchronous and asynchronous remote visits grew and changed the pattern of consultation types during the months of the pandemic. At the end of the study period, non–face-to-face visits gained ground, clearly changing the distribution of the types of visits.

### Evolution of Visits According to the Type of PC Pediatric Services

#### Overview

The data corresponding to the results discussed below are shown in Multimedia Appendix 1, which shows the average daily number of visits in total and by types of visits, separated by month, to PC pediatric services in Catalonia between 2019 and 2022. The change, expressed as a percentage, was calculated with reference to the same period in 2019 and analyzed to see if it was statistically significant.

#### Total Visits

With the emergence of COVID-19 in March 2020, visits decreased significantly from the previous year. It can be observed that the number of daily visits decreased from 15,389 in the month of February 2020 (*P*=.32; vs February 2019) to 9515 in the month of March 2020 (*P*=.001; vs March 2019). The sharpest decrease relative to 2019 was in the month of April 2020 with 5102 visits per day on average (*P*<.001; vs April 2019). This represents a drop of more than 30% of total visits in March and more than 50% in April compared to the same months of the previous year.

In August 2020, compared to August 2019, the number of visits bounced back and the volume of daily visits did not show significant decreases again. In some months it was even significantly higher. The figure for November 2022 stands out, a month in which a maximum of 18,976 visits per day on average was reached (*P*=.002; vs November 2019), which is 40% more visits than the same period of the reference year (Multimedia Appendix 1).

#### Face-to-Face Visits

Regarding face-to-face visits, it can be observed that as of March 2020, there was a significant reduction that persisted until July 2022, the month from which a change in trend was observed and the levels of 2019 were recovered. In April 2020, this decrease was at its maximum and reached 85%, with 1604 visits on average per day compared to 10,987 in April 2019 (*P*<.001). Further on, the reduction was between 48% and 79% throughout 2020, between 17% and 67% in 2021, and between 13% and 47% in 2022, all with respect to 2019 (Multimedia Appendix 1).

#### Synchronous Non–Face-to-Face Visits

As for synchronous non–face-to-face visits, a statistically significant increase was noted starting in March 2020 that was maintained until the end of the study period. March 2020 saw a 200% increase in average daily visits and an increase from 620 daily remote visits in March 2019 to 2073 in March 2020 (*P*<.001). The increase was maintained, and synchronous remote visits went from representing 4.5% of total visits in 2019 to occupying 20% of total visits in 2022 (Multimedia Appendix 1).

#### Asynchronous Non–Face-to-Face Visits

Asynchronous non–face-to-face visits also experienced a statistically significant rise that had already been observed since January 2020 compared to the previous year. While in the first 3 months they had risen by around 60%, from April onward they rose by more than 90%, in May they rose by 154%, and they continued to increase during the following months. Growth continued and asynchronous remote visits increased from 2.8% of total visits in 2019 to 16% of total visits in 2022 (Multimedia Appendix 1).

**Figure 3.**
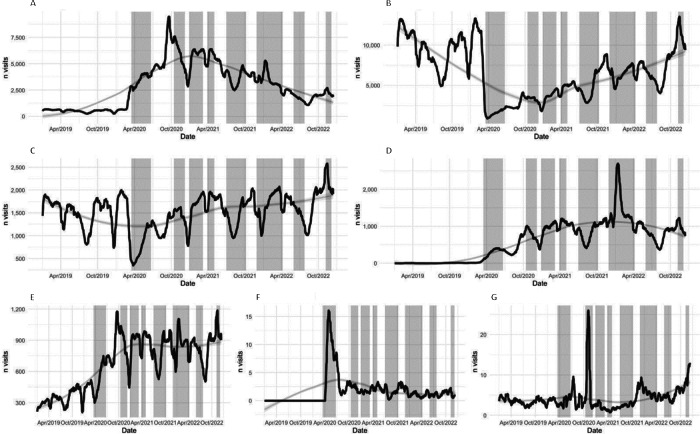
Average daily visits by subtypes to pediatric primary care in Catalonia between January 2019 and December 2022: (A) 9T, (B) 9C, (C) 9R, (D) 9Ec, (E) 9E, (F) 9Ev, and (G) 9D. The vertical stripes indicate the 8 waves of COVID-19 in Catalonia.

Finally, Figure 3 [16,17] shows the evolution of the average number of daily visits according to the different subtypes (9C, 9D, 9R, 9T, 9Ev, 9E, and 9Ec).

Looking at face-to-face visits by subtypes, we can observe a marked reduction in face-to-face visits for acute diseases (9C) starting in March 2020, with a subsequent slow and progressive recovery that did not reach the previous values until November 2022. With respect to the check-ups of the *Infància amb Salut* (9R) program, a significant reduction was also detected as of March 2020, which recovered rapidly and practically completely during the second half of 2020. Pediatric home visits (9D) represented a very small volume with respect to total visits and did not undergo major changes during the years analyzed. However, a spike in these visits was noted during November 2020.

Regarding synchronous remote visits by subtypes, telephone consultations (9T) tripled from February to March 2020 and increased more than tenfold, peaking in September of the same year. Thereafter, a progressive decrease in the number of calls was observed, which at the end of the study period remained above the reference year. As for video call consultations (9Ev), although the values were also very low, we saw the emergence of this type of visit, with a sporadic peak between April and May 2020. Subsequently, they returned to a practically imperceptible level.

Regarding asynchronous remote visits, non–face-to-face visits (9E) already had a progressive growth before the pandemic, but it was between March and September 2020 when the sharpest increase was seen, which was maintained until the end of the period studied. Pediatric eConsultations (9Ec) rose slowly from March 2020 and stabilized at a higher position than before March 2020. A sporadic peak was detected in January 2022.

## Discussion

### Principal Findings

The aim of the study was to analyze the change in the types of pediatric visits to PC centers in Catalonia between 2019 and 2022, following the COVID-19 pandemic. In this context, we have been able to describe the evolution and coexistence between the different types of visits, both face-to-face and non–face-to-face visits, as well as the change in the pattern of pediatric consultations during this period. In summary, the results showed that non–face-to-face visits increased from around 10% of the total in January 2020 (before the pandemic) to almost 25% in December 2022 (after the pandemic). Half of these were telephone consultations and the other half were eConsultations.

Total visits to PC pediatric services fell from March 2020 and did not recover in overall number until September of the same year. From there, the overall volume resumed, with some subsequent fluctuations more or less in line with subsequent waves of COVID-19. In the first wave, there was a marked decrease in face-to-face visits and an increase in teleconsultations, in accordance with the literature. In a study conducted in a PC center in Madrid during the month of June 2020, it was observed that 51% of the visits were remote [18]. Other studies, both nationally and internationally, have also stated that between March and May 2020, the pattern of visits to PC centers changed [19,20]. There was a significant predominance of remote visits, especially by telephone, over face-to-face visits [21].

In the second, third, and fourth waves, face-to-face visits continued to decrease, while remote consultations continued to increase. During the fifth wave, defined by the Delta strain inmid-2021, there was a decline in both face-to-face and remote consultations, possibly because it was not a strain that affected the pediatric age group very much [22]. Nevertheless, remote visits continued to represent a significant part of the total number of visits. This fact changed with the sixth wave, starring the Omicron strain, between December 2021 and February 2022, which particularly affected children [23,24]. The corresponding peak of visits shows that more than half of the consultations at that time were non–face-to-face. During the seventh wave, in mid-2022, no change in the pattern of visits was detected. The eighth wave coincided with the marked increase in total visits, especially face-to-face visits, detected in pediatric services as of November 2022. The considerable increase in visits from November 2022 onward, which exceed prepandemic levels, could be due to the increase in infections reactive to ceasing use of protective measures against COVID-19. This phenomenon has been called immunity debt and may pose a risk for serious epidemics in the near future [25].

A breakdown of visits between face-to-face and non–face-to-face visits clearly shows a drop in the proportion of face-to-face visits and a rise in remote visits as of March 2020. Attendance recovered to prepandemic levels during November 2022 in absolute numbers but not in proportion. Although the number of total visits increased at the end of 2022 compared with 2019, the percentage of face-to-face visits decreased and the percentage of remote visits increased; thus, the results highlight that, during the pandemic, the pattern of the type of pediatric PC visits has changed, as seen in other studies [15,26].

In relation to acute demand (9C), the drop was very sharp, and the recovery was very slow, such that it was not until November 2022, two years later, that levels prior to March 2020 were reached again. This result is similar to the findings of other studies that detected a lower number of visits during the months following the onset of the pandemic, especially in acute infections [27-29]. This may be due to a lower circulation of pathogens other than the SARS-CoV-2 coronavirus. In general, it appears that the reduction in face-to-face attendance did not have much negative impact on urgent pediatric pathology care [30]. However, some studies have reported problems of diagnostic delays, such as an increase in complicated appendicitis cases [31]. On the other hand, an increase in overweight and obesity, technology use, sleep disorders, anxiety and distress, domestic violence, social isolation, and behavioral disturbances were also detected, especially in adolescents [32]. Likewise, due to the reduction in face-to-face attendance, an alteration in the circulation pattern of some respiratory viruses was observed during the subsequent season (2020-2021), especially for respiratory syncytial virus, which had an unusual peak of incidence in midsummer, as reported by the SIVIC [17].

As for the child health check-ups (9R) of the *Infància amb Salut* program, they recovered earlier than visits for acute diseases (9C). Although a drop was detected during March 2020, coinciding with the first wave of COVID-19 and stricter confinement, these visits recovered more quickly and were at the prepandemic level by the end of 2020. Quantification of the delay and losses of check-ups and vaccines showed that vaccination coverage decreased in all the autonomous communities of Spain by between 5% and 60%, depending on age and the type of vaccine [33]. The Spanish Society of Immunology, the Spanish Society of Paediatric Infectious Diseases, and the Spanish Association of Paediatrics published a document in April 2020 with complete information on the decreased uptake of vaccines, along with the criteria for the prioritization of childhood vaccination during the state of alarm carried out by the Ministry of Health [34]. Following the publication of similar figures, European pediatric societies called for the immediate recovery of vaccination programs, since compliance with the established vaccination schedule is one of the prerequisites for dealing with the resulting problems, such as immunity debt and the decreased uptake of vaccines [35].

Telephone consultations (9T), prior to March 2020, represented a minimal volume of total pediatric consultations. The onset of COVID-19 led to an exponential increase in the number of such consultations. They peaked during September 2020, probably coinciding with the start of that year’s school year. Later, an oscillation in the number of calls was observed, although they remained at a higher value than in the period prior to the study. Therefore, the results suggest that telephone consultations have been one of the most widely used tools, both by users and professionals, to replace face-to-face visits during the pandemic [36]. This growth could be due to the ease of use of the telephone as well as to the organization of visits in PC centers, where it was established that the first patient assessment should always be made by telephone [37].

Regarding video call consultations (9Ev), the results have shown that they were used very little during the first wave of home confinement, and this is in agreement with the literature [38]. This low use could be explained by the limited technological resources for video visits, the technical difficulty it could pose for patients with lower digital literacy, and the fact that there was an impression that they did not add value to telephone consultations or eConsultations. Although they are currently practically not used in PC, the literature shows that they have a potential yet to be explored [39,40]. A systematic review conducted in 2021 on telemedicine in pediatrics, which includes 11 studies, concluded that care through call and video-call type visits in various pathologies (obesity, asthma, mental disorders, otitis media, and skin disease) can be comparable to, and in some cases even better than, face-to-face care; for these cases, it is necessary to improve user access and the effectiveness of services [41].

As for telemedicine consultations (9E), which are conducted without the patient being present, they were already on the rise before the pandemic but skyrocketed as of March 2020. One possible explanation is that since most of the COVID-19 cases in pediatrics were mild, many visits were probably made remotely, to indicate the result of the rapid viral detection test to administrative personnel, without ever having contact with health care personnel.

The pandemic was a clear catalyst for the use of eConsultation (9Ec). High use figures were reached and maintained at the end of the study period. In pediatrics, a peak of such visits was detected during January 2022, coinciding with the Omicron wave, which affected children in large numbers but had with low morbidity. Thus, the results of this paper and the literature suggest that eConsultation is here to stay [42].

It is also worth noting that although COVID-19 was a catalyst for the use of telemedicine in clinical practice, the Catalan health system has been promoting digital transformation through various “health plans” and different initiatives since 2011 [43-45]. In this context, the use of teleconsultation during the lockdown was essential to manage the health emergency at that moment. However, the Catalan health system had the digital infrastructure to cope with the situation and provide assistance. The population’s experience of teleconsultation during the lockdown may have an impact on them, and the results also showed that currently, almost 25% of people used teleconsultation after the pandemic period.

During the COVID-19 pandemic, other modalities of pediatric telemedicine care for parents of patients were tested with good resolution and acceptance results, such as the creation of a Twitter (subsequently rebranded as X) profile called Equipo Pediatría HCSC (@EquipoHCSC) by a trained team of pediatricians in Madrid that resolved questions through private messages [46]. Another example is that of a health center in Zaragoza that had already developed an email consultation process, and they observed that during the strictest confinement, its use increased to the detriment of face-to-face consultations, although the most used modality was the telephone [47].

In relation to the use of information and communication technologies in pediatrics, there is the advantage, in general, that there is less of a digital divide on the part of users than in other areas. Several studies indicate that this is due to the age and education that parents usually have [48,49].

It was not only in pediatrics that a sharp reduction in face-to-face visits and a parallel growth in remote visits were detected. Equivalent results have been obtained from family and community medicine [50,51]. Telemedicine, however, has limitations in the human, technological, and economic spheres, and these must be understood by all parties involved. In addition, it is subject to current legislation based on the Organic Law on Data Protection and the doctor-patient confidentiality relationship. Thus, telehealth programs depend largely on the health care setting in which they are implemented. Conditions may change after the pandemic, and further studies will be needed [52].

In short, although the absolute number of face-to-face visits has recovered throughout the pandemic, telemedicine consultations established themselves as an important type of visit and made up almost a quarter of daily visits by the end of 2022. This indicates that some consultations that were previously carried out in person are now likely to be done remotely.

This analysis may contribute to a reorganization of the current model of PC pediatric services, which has been affected for years by the deficit and heterogeneous territorial distribution of professionals [53]. It can also help develop evidence-based guidelines for pediatric remote care focused on accessibility, quality, equity, and efficiency, as has been done in other territories [54,55]. It remains to be seen how, from now on, the application of digital health tools is managed in the field of PC pediatric services in Catalonia, as well as at a more global level, and whether it contributes to improving the current organization.

### Limitations

This study has certain limitations that should be taken into consideration. First, the study did not examine the quality of care or patient outcomes that may have been associated with the changes in PC visits. Therefore, it is not possible to make conclusions about the overall impact of the changes on patient health. Second, the study did not take into account non–COVID-19–related factors that may have influenced PC visits during the pandemic. These factors include changes in patient behavior, such as reluctance to seek medical care due to the fear of contracting COVID-19, or changes in health care provider availability due to workforce shortages or redeployment to COVID-19 care. These factors may have contributed to the observed changes in PC visits and should be considered in future studies.

### Conclusions

The COVID-19 pandemic has been a period of transition for the types of visits to PC pediatric services in Catalonia. It has undeniably accelerated the adoption of telemedicine in various health care systems, including pediatric care in Catalonia. In this context, although face-to-face consultations have recovered in absolute numbers, after the pandemic period, it is evident that telemedicine has gained prominence and plays a significant role in health care delivery. Thus, digital health tools are becoming a real possibility within current pediatric care, and it is difficult to imagine care without the technological integration acquired during the pandemic.

## Supplementary material

10.2196/49943 Multimedia Appendix 1 Average daily visits in total and for each type of visit (face-to-face, synchronous non–face-to-face, and asynchronous non–face-to-face), separated by month, to primary care pediatric services in Catalonia between January 2019 and December 2022. Percentage change with respect to the same period in 2019, SD, and *P* value are also shown.

## Abbreviations

PC: primary care
SIVIC: Sistema d’Informació per a la Vigilància d’Infeccions a Catalunya (Information System for the Surveillance of Infections in Catalonia)
